# Recent advancements in QuIC-based diagnostic assays for sporadic and inherited prion diseases: focusing on the emerging role of ES-QuIC

**DOI:** 10.3389/fnins.2026.1919876

**Published:** 2026-09-14

**Authors:** Rebecca Fox, Jennifer Myskiw, Ben A. Bailey-Elkin, Stephanie A. Booth

**Affiliations:** 1 High Consequence Pathogens Division, National Microbiology Laboratory, Public Health Agency of Canada, Winnipeg, MB, Canada; 2 Department of Medical Microbiology and Infectious Diseases, Rady Faculty of Health Sciences, University of Manitoba, Winnipeg, MB, Canada

**Keywords:** CJD, FFI, IPDs, prion, PRNP, QuIC, TSEs

## Abstract

Prion diseases are a group of fatal neurological disorders characterized by the accumulation of misfolded prion protein, primarily concentrated in the brain. The development of a family of assays known as Quaking-Induced Conversion (QuIC), particularly Real Time (RT)-QuIC, has revolutionized prion disease diagnosis by detecting and amplifying minute quantities of misfolded prion protein into measurable signals. Despite these advancements, certain prion subtypes, such as rare inherited forms or atypical sporadic variants, can produce structural configurations that resist amplification. To address these limitations, we developed a modified QuIC-based assay to increase sensitivity across a wider spectrum of prion diseases. Through retrospective analysis of cerebrospinal fluid (CSF) and brain tissue samples from patients diagnosed with both sporadic Creutzfeldt–Jakob disease (sCJD) and inherited prion diseases (IPDs), many of them historically problematic for conventional QuIC testing, we benchmark this modified assay against its predecessors. The updated assay demonstrated more consistent detection of disease-associated prion protein, successfully identifying cases that had previously yielded negative, inconclusive or borderline results. Given the transmissible nature of prion diseases, early and accurate diagnostic detection carries considerable weight in guiding patient care and counseling, as well as in broader public health surveillance. Taken together, these results demonstrate the strong diagnostic potential of this enhanced assay and support further prospective and independent evaluation toward its incorporation into standard diagnostic workflows for patients with suspected prion disease.

## Introduction

Prion diseases belong to a broader category of diseases known as proteinopathies, conditions driven by the accumulation of misfolded forms of naturally occurring monomeric proteins into abnormal, self-propagating configurations (Barron, 2017; Bayer, 2015). Prion diseases are specifically defined by the accumulation of misfolded prion proteins (PrP) (Barron, 2017), which form structured aggregates known as amyloid fibrils that develop through a process of seeded polymerization (Ma and Wang, 2014).

The detailed structures of some of these fibrils have been identified through cryo-electron microscopy, demonstrating structurally distinct conformers dependent on variants of the disease (Manka et al., 2023). The growth of these fibrils occurs through continuous, templated addition of soluble protein to the ends of existing amyloid seeds (Rhoads et al., 2020). This templating behavior is the basis for the groundbreaking prion detection method called quaking-induced conversion (QuIC) (Slota et al., 2025; Wilham et al., 2010).

Quaking-induced conversion-based assays use intervals of vigorous shaking to fragment growing amyloid fibrils and increase the number of active templating ends. Recombinant protein substrate is provided as raw material for extension and elongation of new fibril growth, which is detected by fluorescence with the addition of the amyloid-specific dye, Thioflavin T (ThT) (Orrú et al., 2015). Repeated cycles of shaking and extension can amplify prion seeds up to a trillion-fold, making it possible to detect prions in clinical and environmental samples with remarkable sensitivity (Rhoads et al., 2020; Slota et al., 2025).

Although QuIC has revolutionized prion testing by enabling detection from readily accessible patient material such as cerebrospinal fluid (CSF), certain disease subtypes remain difficult to detect (Poleggi et al., 2022). This may be due, in part, to structural differences in their oligomeric and fibrillar forms that result in reduced seeding efficiency and poorer amplification potential. In this review, we provide a comprehensive summary of the performance of CSF-based QuIC testing, encompassing reported sensitivity and specificity across various clinical contexts. Our laboratory has served as Canada’s national diagnostic laboratory for CSF-based CJD testing using the ISO-accredited end point-QuIC (EP-QuIC) assay for the past 8 years, with its performance validated against neuropathological confirmation in up to 60% of Canadian CJD cases during this period (Slota et al., 2025). Building on this established diagnostic platform, this review also examines our recently developed Enhanced Sensitivity QuIC (ES-QuIC), which retains the EP-QuIC methodology while demonstrating improved detection of prion disease subtypes that have historically been difficult to identify using QuIC-based seeding assays.

## Classification of human prion diseases

The majority of human prion disease cases detected in the clinic occur sporadically, which consistently account for approximately 85%–90% of detected cases globally, and arise without an identifiable cause (Gambetti et al., 2003). Of this classification, sporadic Creutzfeldt–Jakob disease (sCJD) represents the most prevalent form, alongside rarer entities such as sporadic fatal insomnia (sFI), and variably protease-sensitive prionopathy (VPSPr) (Brown, 2001; Nathanson et al., 1997; Poleggi et al., 2022; Will, 2003). The remainder of cases (∼15%) generally fall within the category of inherited prion diseases (IPDs), except for acquired CJD cases, which occur rarely as iatrogenic transmissions or historical outbreaks as in the case of bovine spongiform encephalopathy or kuru (Brown, 2001; Nathanson et al., 1997; Will, 2003).

Polymorphisms of the *PRNP* gene, which encodes the cellular prion protein, can dictate the disease phenotype of sCJD and IPDs depending on whether the patient is homo- or heterozygous for methionine or valine (MM, VV, or MV) at codon 129 (Appleby et al., 2022; Nathanson et al., 1997; Zerr et al., 2024). When combined with the biochemical characteristic of the proteinase K resistant core of misfolded prion proteins, six primary molecular subtypes are resolved MM1, MM2, MV1, MV2, VV1, and VV2 (Chen and Dong, 2016; Nathanson et al., 1997; Zerr et al., 2024). Further correlation with clinical presentation and neuropathology can reveal distinct phenotypic associations, and cases can often be classified further within the following histopathological subtypes: MM/MV1, VV2, MV2K, MM2C, MM2T, and VV1 (Poleggi et al., 2022; Zerr et al., 2024).

Additional diversity is apparent when IPDs are considered (Appleby et al., 2022; Chen and Dong, 2016; Collinge, 2001). More than 60 different genetic mutations have been characterized, that vary in clinical presentation, neuropathology and in their biochemical profiles (Appleby et al., 2022). A number of these IPDs, including fatal familial insomnia (FFI) and Gerstmann-Stäussler-Scheinker disease (GSS), are particularly insensitive to detection in CSF samples by most QuIC-based methods (Gambetti et al., 2003). The D178N mutation is responsible for both FFI and gCJD phenotypes, determined by its location alongside the M or V codon 129 allele of *PRNP*, respectively (Goldfarb et al., 1992; Synofzik et al., 2009). These ongoing challenges in detecting certain sporadic and inherited forms of CJD from ante-mortem CSF highlight the ongoing need for further technical innovation. Advancements are not only necessary to broaden the range of detectable prion subtypes, but also to establish consistent parameters that can be standardized and reliably applied across different laboratory settings. Consequently, QuIC-based assays have emerged as the foundation of ante-mortem prion disease diagnosis, providing the platform upon which many recent methodological improvements have been developed.

## QuIC testing for human prion diseases

Quaking-induced conversion testing of CSF obtained via lumbar puncture has become a primary pre-mortem diagnostic testing method, which, in conjunction with a clinical phenotype of a progressive neuropsychological syndrome, can accurately identify most human prion diseases (Hermann et al., 2018). Importantly, QuIC testing varies in implementation across international diagnostic and surveillance laboratories. While most laboratories use QuIC in its real-time (RT) format, in Canada, the diagnostic standard uses an end point (EP) format (Simon et al., 2021; Slota et al., 2025).

The primary differences between RT- and EP-QuIC lie in the instruments used for sample agitation and the timing of fluorescence measurements. For RT-QuIC, fluorescence intensity is measured every 15 min with cycles of agitation at 60 s (700 rpm, double-orbital) and 60 s rest (Orrú et al., 2014, 2015). The positivity threshold is calculated as an RFU exceeding the mean fluorescence measured between 30 and 90 min plus five standard deviations when using full-length Syrian hamster PrP (Rhoads et al., 2020; Slota et al., 2025). In contrast, EP-QuIC agitates the plate in cycles of 90 s (900 rpm, orbital) followed by 30 s rest. Sample fluorescence is not measured continuously; instead, an initial measurement is taken at 20 min and a final measurement at 66 h using a suitable fluorescence reader (McGuire et al., 2012; Myskiw et al., 2026; Slota et al., 2025). For EP-QuIC, the diagnostic positivity threshold is defined as an RFU at 66 h that is greater than four times the baseline fluorescence measured at 20 min (Rhoads et al., 2020; Slota et al., 2025).

The recombinant prion protein used as QuIC substrate is most commonly either full length (1*^st^* generation) or truncated (2*^nd^* generation) Syrian Hamster PrP (HaPrP_23–231_ and HaPrP_90–231_, respectively). Whilst 2*^nd^* generation RT-QuIC is more frequently used across diagnostic laboratories and surveillance programs worldwide, EP-QuIC has demonstrated reliable performance and reduced non-specific seeding activity occasionally observed with 2*^nd^* generation testing. In the first 8 years of use, the sensitivity and specificity were assessed at 96.7% and over 99%, respectively, using autopsy-confirmed cases that represented more than 50% of cases identified in Canada during this period (Slota et al., 2025). For cross-study comparisons, the sensitivity and specificity of various recombinant PrP substrates used in CSF testing for CJD across international sites and different QuIC methods are consolidated in Table 1.

**TABLE 1:** Summary of sensitivity and specificity for detection of prion protein (PrP) in CSF samples using various recombinant PrP strains and generations of QuIC.

Reference	Recombinant PrP	Diagnosis group (*n*. of cases)	Subtype (*n*. of cases)	Sensitivity (%) (95% CI)	Controls (*n*. of cases)	Specificity (%) (95% CI)
1st Generation Real Time-QuIC						
Atarashi et al., 2011	Hu PrP23-231	CJD (18)	–	83.3 (58.6–96.4)	CTRL (35)	100.0 (90.0–100.0)
		CJD (16)	–	87.5 (61.7–98.4)	OND (144)	100.0 (97.5–100.0)
Correia et al., 2026	Hu PrP23-231	sCJD (54)	MM/MV1 (+2C) (43)	76.7 (61.4–88.2)	Non-CJD (62)	100.0 (94.2–100.0)
			VV2 (8)	87.5 (47.3–99.7)		
			MV2K (3)	100.0 (29.2–100.0)		
		gCJD (18)	E200K (18)	83.3 (58.6–96.4)		
		FFI (22)	D178N (22)	36.4 (17.2–59.3)		
		GSS (2)	–	50.0 (1.3–98.7)		
	Hu-E200K PrP23-231	sCJD (67)	MM/MV1 (+2C) (43)	97.7 (87.7–99.9)	Non-CJD (62)	100.0 (94.2–100.0)
			VV2 (8)	100.0 (63.1–100.0)		
			MV2K (3)	100.0 (29.2–100.0)		
			MM/MV2C (13)	61.5 (31.6–86.1)		
		gCJD (18)	E200K (18)	100.0 (81.5–100.0)		
		FFI (32)	D178N (32)	75.0 (56.6–88.5)		
		GSS (3)	–	100.0 (29.2–100.0)		
	Atypical sCJD (8)	VV1 (8)	87.5 (47.3–99.7)			
Park et al., 2016	Hu PrP24-234	sCJD (81)	–	76.5 (65.8–85.2)	Non-CJD (100)	100.0 (96.4–100.0)
Cramm et al., 2016	HaSh PrP14-234	sCJD (64)	–	80.0 (67.7–88.7)	CTRL (400)	99.0 (97.5–99.7)
		gCJD (39)	E200K (33)	100.0 (89.4–100.0)		
			V210I (6)	100.0 (54.1–100.0)		
		FFI (7)	D178N (7)	57.0 (18.4–90.1)		
Hermann et al., 2018	HaSh PrP14-234	sCJD (65)	–	89.0 (79.1–95.6)	Non-CJD (118)	100.0 (96.9–100.0)
Hermann et al., 2023	HaSh PrP14-234	sCJD (161)	MM/MV1 (94)	88.3 (80.0–94.0)	Non-CJD (371)	98.7 (96.9–99.6)
			MM/MV 1 + 2C (23)			91.0 (72.0–98.9)
			VV2 (19)			100.0 (82.4–100.0)
			MV2K (12)			92.0 (61.5–99.8)
			MM/MV2C (8)			63.0 (24.5–91.5)
			VV1 (5)			60.0 (14.7–94.7)
			VV2 (4)			100.0 (39.8–100.0)
			MV1 (3)			100.0 (29.2–100.0)
			MV2 (3)			100.0 (29.2–100.0)
		Atypical sCJD (1)	VPSPr (1)			100.0 (2.5–100.0)
		gCJD (2)	E200K (2)			100.0 (15.8–100.0)
		GSS (2)	P102L (2)			100.0 (15.8–100.0)
Correia et al., 2026	HaSh PrP14-234	sCJD (67)	MM/MV1 (+2C) (43)	83.7 (69.3–93.2)	Non-CJD (62)	100.0 (94.2–100.0)
			VV2 (8)	75.0 (34.9–96.8)		
			MV2K (3)	100.0 (29.2–100.0)		
			MM/MV2C (13)	53.8 (25.1–80.8)		
		gCJD (18)	E200K (18)	83.3 (58.6–96.4)		
		FFI (32)	D178N (32)	18.8 (7.2–36.4)		
		GSS (3)	–	33.3 (0.8–90.6)		
		Atypival sCJD (8)	VV1 (8)	37.5 (8.5–75.5)		
Mok et al., 2021	Bv PrP23-231	sCJD (79)	–	88.6 (79.5–94.7)	Non-CJD (57)	91.2 (80.7–97.1)
		vCJD (2)	–	50.0 (1.3–98.7)		
		gCJD (20)	D178N (2)	50.0 (1.3–98.7)		
			E196K (1)	100.0 (2.5–100.0)		
			E200K (6)	100.0 (54.1–100.0)		
			4 OPRI (2)	0.0 (0.0–84.2)		
		GSS (11)	6 OPRI (4)	75.0 (19.4–99.4)		
			P102L (5)	20.0 (0.5–71.6)		
			A117V (2)	0.0 (0.0–84.2)		
		PrP-CAA (1)	Y163X (1)	0.0 (0.0–97.5)		
Myskiw et al., 2026	Dm PrP23-230	sCJD (61)	–	83.6 (71.9–91.8)	Non-CJD (71)	97.1 (90.2–99.7)
McGuire et al., 2012	Ha PrP23-231	sCJD (123)	–	89.0 (81.6–93.6)	Non-CJD (103)	99.0 (94.7–100.0)
Orrú et al., 2014	Ha PrP23-231	sCJD (28)	MM (14)	78.6 (49.2–95.3)	Non-CJD (46)	100.0 (92.3–100.0)
			MV (8)	75.0 (34.9–96.8)		
			VV (2)	100.0 (15.8–100.0)		
			Unknown (4)	75.0 (19.4–99.4)		
		gCJD (2)	E200K (2)	50.0 (1.3–98.7)		
Groveman et al., 2016	Ha PrP23-231	CJD (113)	–	73.0 (63.4–80.5)	Non-CJD (64)	100.0 (94.4–100.0)
Lattanzio et al., 2017	Ha PrP23-231	sCJD (305)	MM (190)	84.2 (78.2–89.1)	Non-CJD (348)	99.4 (97.9–99.9)
			MV (72)	72.2 (60.4–82.1)		
			VV (44)	79.5 (64.7–90.2)		
		gCJD (46)	E200K -129M (16)	100.0 (79.4–100.0)		
			V210I – 129M (21)	95.2 (76.2–99.9)		
			E200K -129V (4)	100.0 (39.8–100.0)		
			4 OPRI -129M (1)	100.0 (2.5–100.0)		
			D178N -129V (2)	0.0 (0.0–84.2)		
			R208H -129V (1)	0.0 (0.0–97.5)		
			V203I -129M (1)	100.0 (2.5–100.0)		
		Atypical sCJD (1)	VPSPr (1)	0.0 (0.0–97.5)		
Bongianni et al., 2017	Ha PrP23-231	sCJD (50)	–	72.0 (57.5–83.8)	Non-CJD (0)	–
		gCJD (8)	–	50.0 (15.7–94.3)		
Rudge et al., 2018	Ha PrP23-231	sCJD (75)	–	89.0 (80.1–95.3)	Non-CJD (64)	100.0 (94.4–100.0)
Abu-Rumeileh et al., 2019	Ha PrP23-231	sCJD (80)	MM(V)1 (39)	82.1 (66.5–92.5)	Non-CJD (109)	100.0 (96.7–100.0)
			VV2 (19)	94.7 (74.0–99.9)		
			MV2K (16)	75.0 (47.6–92.7)		
			MM2C (4)	75.0 (19.4–99.4)		
			MM2T (1)	0.0 (0.0–97.5)		
			VV1 (1)	100.0 (2.5–100.0)		
		Atypical sCJD (1)	VPSPr (1)	0.0 (0.0–97.5)		
		gCJD (22)	E200K (11)	100.0 (71.5–100.0)		
			V210I (8)	100.0 (63.1–100.0)		
			D178N (1)	0.0 (0.0–97.5)		
			R208H (1)	100.0 (2.5–100.0)		
			E219G (1)	0.0 (0.0–97.5)		
Myskiw et al., 2026	Ha PrP23-231	sCJD (61)	–	83.6 (71.9–91.8)	Non-CJD (71)	97.1 (90.2–99.7)
2nd Generation Real Time-QuIC						
Orrú et al., 2015	Ha PrP90-231	sCJD (48)	MM1 (18)	94.4 (72.7–99.9)	Non-CJD (39)	100.0 (91.0–100.0)
			MV1 (7)	100.0 (59.0–100.0)		
			MV2 (5)	100.0 (47.8–100.0)		
			VV2 (7)	100.0 (59.0–100.0)		
			Unknown (7)	85.7 (42.1–99.6)		
			MM (2)	100.0 (15.8–100.0)		
			MV (1)	100.0 (2.5–100.0)		
			VV (1)	100.0 (2.5–100.0)		
Groveman et al., 2016	Ha PrP90-231	CJD (113)	MM	98.0	Non-CJD (64)	100.0 (94.4–100.0)
			MV	96.0		
			VV	90.0		
Franceschini et al., 2017	Ha PrP90-231	sCJD (116)	MM1 (43)	93.0 (80.9–98.5)	Non-CJD (100)	100.0 (96.4–100.0)
			VV2 (33)	100.0 (89.4–100.0)		
			MV2K (26)	92.3 (74.9–99.1)		
			MM2C (9)	66.7 (29.9–92.5)		
			MM2T (4)	75.0 (19.4–99.4)		
			VV1 (1)	100.0 (2.5–100.0)		
		gCJD (33)	E200K -129M (20)	100.0 (83.2–100.0)		
			V210I – 129M (10)	100.0 (69.2–100.0)		
			D178N – 129V (1)	100.0 (2.5–100.0)		
			R208H – 129V (1)	100.0 (2.5–100.0)		
			E219G -129V (1)	100.0 (2.5–100.0)		
		GSS (6)	P102L (3)	25.0 (0.8–90.6)		
			D202N (1)	0.0 (0.0–97.5)		
			A117V (1)	100.0 (2.5–100.0)		
			8 OPRI (1)	0.0 (0.0–97.5)		
		FFI (2)	D178N (2)	0.0 (0.0–84.2)		
		Atypical sCJD (3)	VPSPr (3)	100.0 (29.2–100.0)		
Bongianni et al., 2017	Ha PrP90-231	sCJD (22)	–	86.4 (65.1–97.1)	Non-CJD (17)	100.0 (80.5–100.0)
		gCJD (2)	–	50.0 (1.3–98.7)		
Foutz et al., 2017	Ha PrP90-231	sCJD (63)	MM1 (29)	96.6 (82.2–99.9)	RPD (14)	100.0 (76.8–100.0)
			MV1 (5)	80.0 (28.4–99.5)		
			VV2 (11)	100.0 (71.5–100.0)		
			MM2C (2)	100.0 (15.8–100.0)		
			MM1-2 (6)	100.0 (54.1–100.0)		
			MV1-2 (8)	100.0 (63.1–100.0)		
			VV1-2 (1)	100.0 (2.5–100.0)		
			sFI (1)	0.0 (0.0–97.5)		
		gCJD (2)	–	100.0 (15.8–100.0)		
		sCJD (111)	MM1 (61)	95.1 (86.3–99.0)	RPD (67)	98.5 (92.0–100.0)
			MV1 (10)	100.0 (69.2–100.0)		
			MV2 (9)	88.9 (51.8–99.7)		
			VV1 (8)	75.0 (34.9–96.8)		
			VV2 (15)	100.0 (78.2–100.0)		
			MM2-C (7)	71.4 (29.0–96.3)		
			sFI (1)	0.0 (0.0–97.5)		
		gCJD (12)	–	100.0 (73.5–100.0)		
		GSS (2)	–	50.0 (1.3–98.7)		
		FFI (1)	D178N (1)	100.0 (2.5–100.0)		
Abu-Rumeileh et al., 2019	Ha PrP90-231	sCJD (62)	MM(V)1 (24)	91.7 (73.0–99.0)	Non-CJD (53)	100.0 (93.3–100.0)
			VV2 (18)	100.0 (81.5–100.0)		
			MV2K (16)	100.0 (79.4–100.0)		
			MM2C (2)	100.0 (15.8–100.0)		
			MM2T (1)	100.0 (2.5–100.0)		
			VV1 (1)	100.0 (2.5–100.0)		
		Atypical sCJD (1)	VPSPr (1)	100.0 (2.5–100.0)		
		gCJD (15)	E200K (9)	100.0 (66.4–100.0)		
			V210I (3)	100.0 (29.2–100.0)		
			D178N (1)	100.0 (2.5–100.0)		
			R208H (1)	100.0 (2.5–100.0)		
			E219G (1)	100.0 (2.5–100.0)		
Fiorini et al., 2020	Ha PrP90-231	sCJD (102)	–	96.0 (90.3–98.9)	Non-CJD (80)	100.0 (95.5–100.0)
Rhoads et al., 2020	Ha PrP90-231	sCJD (439)	–	92.9 (90.1–95.2)	CTRL (69)	98.5 (92.2–100.0)
		sFI (5)	–	0.0 (0.0–52.2)		
		Atypical sCJD (3)	VPSPr (3)	66.7 (9.4–99.2)		
		gCJD (31)	–	96.8 (83.3–99.9)		
		FFI (4)	D178N (4)	0.0 (0.0–60.2)		
		GSS (3)	–	33.3 (0.8–90.6)		
Xiao et al., 2020	Ha PrP90-231	sCJD (30)	–	96.7 (82.8–99.9	Non-CJD (30)	100.0 (88.4–100.0)
Myskiw et al., 2026	Ha PrP90-231	sCJD (61)	–	95.1 (86.3–99.0)	Non-CJD (71)	95.8 (88.1–99.1)
		gCJD (15)	E200K (7)	100.0 (59.0–100.0)		
			V210I (2)	100.0 (15.8–100.0)		
			V180I (1)	100.0 (2.5–100.0)		
			D178N (1)	0.0 (0.0–97.5)		
			OPRI (4)	100.0 (39.8–100.0)		
		FFI (13)	D178N (13)	0.0 (0.0–24.7)		
		GSS (12)	P102L (10)	40.0 (12.2–73.8)		
			P105S (1)	0.0 (0.0–97.5)		
			A117V (1)	0.0 (0.0–97.5)		
		Atypical sCJD (15)	VV1 (5)	40.0 (5.3–85.3)		
			MM2 (5)	60.0 (14.7–94.7)		
			VPSPr (5)	40.0 (5.3–85.3)		
End Point-QuIC						
Simon et al., 2021	Ha PrP23-231	CJD (122)	–	95.9 (90.7–98.7)	Non-CJD (501)	99.0 (97.7–99.7)
Slota et al., 2025	Ha PrP23-231	sCJD (274)	MM1 (118)	93.0 (87.1–97.0)	Non-CJD (35)	97.0 (85.1–99.9)
			MM2 (13)	100.0 (75.3–100.0)		
			MM Mixed (17)	100.0 (80.5–100.0)		
			MV1 (32)	97.0 (83.8–99.9)		
			MV2 (28)	93.0 (76.5–99.1)		
			MV Mixed (11)	91.0 (58.7–99.8)		
			VV2 (51)	98.0 (89.6–100.0)		
			VV Mixed (4)	100.0 (39.8–100.0)		
Myskiw et al., 2026	Ha PrP23-231	sCJD (61)	–	100.0 (94.1–100.0)	Non-CJD (71)	100.0 (94.9–100.0)
		gCJD (15)	E200K (7)	100.0 (59.0–100.0)		
			V210I (2)	100.0 (15.8–100.0)		
			V180I (1)	0.0 (0.0–97.5)		
			D178N (1)	100.0 (2.5–100.0)		
			OPRI (4)	100.0 (39.8–100.0)		
		FFI (13)	D178N (13)	30.8 (9.1–61.4)		
		GSS (12)	P102L (10)	80.0 (44.4–97.5)		
			P105S (1)	100.0 (2.5–100.0)		
			A117V (1)	0.0 (0.0–97.5)		
		Atypical sCJD (15)	VV1 (5)	100.0 (47.8–100.0)		
			MM2 (5)	100.0 (47.8–100.0)		
			VPSPr (5)	60.0 (14.7–94.7)		
**Enhanced Sensitivity-QuIC**						
Myskiw et al., 2026	Dm PrP23-230	sCJD (61)	–	100.0 (94.1–100.0)	Non-CJD (71)	100.0 (94.9–100.0)
		gCJD (15)	E200K (7)	100.0 (59.0–100.0)		
			V210I (2)	100.0 (15.8–100.0)		
			V180I (1)	0.0 (0.0–97.5)		
			D178N (1)	100.0 (2.5–100.0)		
			OPRI (4)	100.0 (39.8–100.0)		
		FFI (13)	D178N (13)	92.3 (64.0–99.8)		
		GSS (12)	P102L (10)	100.0 (69.2–100.0)		
			P105S (1)	100.0 (2.5–100.0)		
			A117V (1)	100.0 (2.5–100.0)		
		Atypical sCJD (15)	VV1 (5)	100.0 (47.8–100.0)		
			MM2 (5)	100.0 (47.8–100.0)		
			VPSPr (5)	60.0 (14.7–94.7)		

Reporting criteria for sample positivity and diagnosis varied considerably among laboratories and studies. Direct head-to-head comparisons of assay performance were possible within the study by Myskiw et al. (2026), in which multiple QuIC assay generations were evaluated using the same sample set and therefore under comparable study conditions. QuIC results are grouped according to assay generation, with ES-QuIC highlighted in green to emphasize its promising sensitivity and specificity. Where sufficient numerator and denominator data were available, 95% confidence intervals were calculated using the exact binomial (Clopper–Pearson) method. Confidence intervals were not calculated where the necessary underlying data were unavailable. These estimates are based on the reported study populations and should not be interpreted as pooled estimates of diagnostic performance. Disease and patient groups: CJD, Creutzfeldt–Jakob disease; sCJD, sporadic CJD; atypical sCJD, rare sCJD subtypes including VPSPr, VV1, and MM2; VPSPr, variably protease-sensitive prionopathy; sFI, sporadic fatal insomnia; GSS, Gerstmann–Sträussler–Scheinker syndrome; FFI, fatal familial insomnia; gCJD, genetic CJD; PrP-CAA, prion protein cerebral amyloid angiopathy (Mead et al., 2013); CTRL, control group (not specified); OND, other neurological disorders; non-CJD, patients without CJD; RPD, rapidly progressive dementia. Recombinant substrates: Hu PrP23–231, recombinant human PrP [23–231]; Ha PrP23–231, recombinant full-length Syrian hamster PrP [23–231]; Ha PrP90–231, recombinant truncated Syrian hamster PrP [90–231]; HaSh PrP14–234, recombinant hamster–sheep chimeric PrP [14–234]; Hu PrP24–234, recombinant human PrP [24–234]; Bv PrP23–231, recombinant full-length bank vole PrP [23–231]; Dm PrP23–230, recombinant full-length deer mouse PrP [23–230]. Assays: RT-QuIC, real-time quaking-induced conversion; EP-QuIC, endpoint quaking-induced conversion; ES-QuIC, enhanced sensitivity quaking-induced conversion.

Although definitive diagnosis of sCJD requires post-mortem neuropathological or biochemical analysis of brain tissue, declining autopsy rates and reductions in public health funding make this increasingly impractical (Coulthart et al., 2015; Lillquist et al., 2006; Louie et al., 2004). Clinical assessment alone is complicated by the frequency with which sCJD phenotypically resembles other, more common neurological conditions, particularly in early stages of disease (Poleggi et al., 2022; Shimamura and Satoh, 2025). Timely advances in ante-mortem testing have helped mitigate these diagnostic challenges; however, important limitations remain in the detection of certain prion disease subtypes and IPDs.

## Advancements in detection of sporadic and inherited human prion diseases

Despite substantial progress in prion disease testing, several IPDs and some rare sCJD subtypes such as VV1 and MM2 remain challenging to detect. Importantly, direct comparisons between studies are complicated by the rarity of these diseases, which have limited the number of large cohorts evaluated using QuIC assays. FFI and GSS, in particular, are consistently associated with reduced sensitivity to RT-QuIC (Correia et al., 2026; Foutz et al., 2017; Schmitz et al., 2022). Consequently, these cases may remain unrecognized until post-mortem analysis. More specifically, 1*^st^* generation RT-QuIC has limited sensitivity for FFI and some gCJD phenotypes associated with the D178N mutation (Lattanzio et al., 2017). Although 2nd generation RT-QuIC has improved diagnostic sensitivity toward sCJD, these improvements have not extended to IPDs with non-CJD phenotypes, such as FFI and GSS (Abu-Rumeileh et al., 2019; Correia et al., 2026; Mok et al., 2021; Rhoads et al., 2020).

Recombinant substrates used in diagnostic QuIC assays have predominantly been derived from rodent *PRNP* sequences, reflecting their extensive use as animal models for prion disease and their ability to support prion replication. These substrates include the previously mentioned Syrian Hamster PrP sequence, as well as that of the bank vole, which is often described as a “universal acceptor” due to its susceptibility to a broad range of prion strains (Nonno et al., 2006; Watts et al., 2014). Modified substrates have also been explored, including the truncated Syrian Hamster construct used in 2*^nd^* generation RT-QuIC, which lacks a large portion of the N-terminal unstructured region and produces shorter lag-times compared to the full-length counterpart (Wilham et al., 2010). Species-matched substrates have also been investigated based on the assumption that greater sequence identity with the prion seed may enhance seeding efficiency by providing a more compatible template. For example, human PrP variants carrying disease-associated mutations, such as E200K, have been used to detect seeding activity in human clinical samples with low seed abundance (Schmitz et al., 2023).

Despite continued development of QuIC, the factors governing the relationship between seeding efficiency and substrate remain incompletely understood. Importantly, an optimal QuIC should enable robust detection of diverse prion seeds at low abundance, while maintaining sufficient conformational stability in the absence of seeds to minimize spontaneous conversion and reduce false-positive signals. Our laboratory recently developed Enhanced Sensitivity QuIC (ES-QuIC), which uses recombinant substrate derived from the North American deer mouse (*Peromyscus maniculatus*) *PRNP* sequence (Myskiw et al., 2026). The positivity threshold for this assay is defined as an RFU exceeding the mean baseline fluorescence measured at 20 min by more than 20 standard deviations, with positive reactions reaching the threshold between 50 and 66 h (Myskiw et al., 2026). ES-QuIC has significantly improved diagnostic performance toward inherited and atypical prion diseases, as outlined in Table 1 (Myskiw et al., 2026).

Using ES-QuIC, the available evidence suggests high sensitivity for detection of FFI in a cohort of 13 CSF samples, with 12 of 13 samples testing positive and the remaining sample consistently producing an indeterminate result (Correia et al., 2026; Myskiw et al., 2026). The remaining IPDs in our retrospective cohort demonstrated a sensitivity of 100%, except for a single case with a V180I mutation. We also observed 100% detection of the rare sCJD subtypes VV1 and MM2, and a sensitivity of 60% for the atypical prion disease VPSPr. These findings suggest that the sensitivity gains observed with ES-QuIC are not limited to FFI cases and may extend to other prion disease phenotypes that have historically been difficult to detect using QuIC. However, the reduced sensitivity observed for VPSPr underscores the continued challenge of detecting protease-sensitive prion disease subtypes.

The development of ES-QuIC aligns with ongoing efforts to optimize recombinant protein substrate design and improve QuIC sensitivity for prion disease variants that have proven difficult to detect. Several recently published studies using human *PRNP*-derived substrates have also reported promising sensitivity gains in QuIC assays. For example, Correia et al. (2026), reported good performance using Hu-E200K PrP_23–231_ in 1*^st^* generation RT-QuIC, with both sCJD, gCJD, and GSS cases. A substrate based on the E219K polymorphism has demonstrated amplification in six sCJD strains as well as vCJD, but showed limited sensitivity for detecting the VV1 prion strain in a patient-derived sample (Marín-Moreno et al., 2025). These findings prompted us to develop a method for directly comparing the seeding potential of various substrates across panels of IPD and CJD subtypes. We compared seeding efficiency among recombinant prion substrates, including full-length and truncated Syrian Hamster, deer mouse, and E200K, using log dilutions of brain homogenates as seeds (Myskiw et al., 2026). The limit of detection provided a direct comparison of seeding that correlated well with the relative sensitivities observed in CSF testing. The Dm PrP_23–230_ substrate demonstrated higher reported sensitivity than the full-length and truncated hamster substrates and the E200K substrate in this study (Myskiw et al., 2026). Moreover, the poor performance of truncated hamster substrate for detecting various IPDs was evident in this assay, mirroring the findings from CSF testing (Myskiw et al., 2026). These data support the use of this type of assay for screening novel substrates.

Interestingly, Schmitz et al. (2023) reported that, using the E200K substrate in an analysis of tear fluid, seeding activity was detected not only in symptomatic individuals with the E200K mutation, but also in four out of five asymptomatic mutation carriers, at time-points that were not specific to clinical symptom onset. These findings raise the possibility that positive seeding signals obtained with human-derived recombinant substrates may reflect amplification of misfolded mutant PrP species in intermediate conformations, rather than exclusively indicating the presence of infectious prion seeds (Wang et al., 2017). However, alternative explanations should be considered, including spontaneous conversion of the recombinant substrate, genuine prion seeding present during the preclinical stage, or other non-specific amplification events.

We acknowledge several inherent limitations of ES-QuIC, including the technical challenges associated with reproducing the deer mouse recombinant substrate, such as batch-to-batch variability in its production, as well as spontaneous conversion and background fluorescence. To mitigate these challenges, extensive quality control measures are used to determine whether a lot is acceptable, including SDS-PAGE, differential scanning fluorimetry (DSF), and QuIC using standardized positive and negative controls, followed by a negative control panel and a mutation panel. To support continued assay development, ES-QuIC is currently undergoing preliminary parallel evaluation with EP-QuIC using routine diagnostic CSF samples. This ongoing evaluation will provide additional evidence regarding assay robustness, reproducibility, and the frequency of spontaneous substrate conversion, while also facilitating direct comparison with an established diagnostic platform. Parallel testing is particularly important given the limited availability of IPD cases including rare GSS-associated mutations and the scarcity of subtypes such as VV1 and VPSPr, which were underrepresented in our initial retrospective study. However, further prospective evaluation with appropriate clinical and neuropathological follow-up is required to establish its diagnostic performance and clinical utility. As additional diagnostic samples are accrued, these analyses will help determine whether the improved sensitivity of ES-QuIC can be consistently maintained without compromising specificity across broader patient populations and independent laboratory settings. Although the assay shows promising performance overall, its reduced sensitivity for VPSPr suggests that negative results should be interpreted with caution in patients with clinical features suggestive of protease-sensitive prion diseases. Despite these limitations, ES-QuIC has demonstrated detection of prion disease associated with the D178N mutation and GSS in the cohorts evaluated to date, supporting its potential as a next-generation assay for ante-mortem detection of prion seeding activity across a broad spectrum of human prion diseases (Myskiw et al., 2026).

## Discussion

Quaking-induced conversion-based assays continue to advance the ante-mortem diagnosis of human prion diseases, offering high sensitivity and specificity for the majority of sporadic CJD cases and many inherited forms. However, persistent diagnostic gaps in atypical sCJD subtypes and several IPDs underscore the need for ongoing refinement of assay conditions and substrate selection. The development of ES-QuIC, together with emerging human *PRNP*-derived substrates, demonstrates that exploring the use of different substrates, along with optimizing quaking dynamics, may improve detection of challenging phenotypes. Comparative seeding analyses further support the utility of systematic substrate evaluation as a means of predicting diagnostic performance across diverse prion strains (Myskiw et al., 2026; Slota et al., 2025). Moreover, ES-QuIC demonstrated high sensitivity in the human prion disease cohorts evaluated to date and is amenable to diagnostic implementation. Once recombinant substrate is available, the assay requires relatively inexpensive instrumentation, and positivity criteria can be applied using predefined thresholds that may facilitate standardized implementation and test accreditation. A common concern with highly sensitive amplification assays is the potential trade-off between sensitivity and specificity. In our retrospective cohort, ES-QuIC demonstrated improved sensitivity while maintaining 100% observed specificity, with all non-CJD samples correctly classified as negative, including samples from individuals with Alzheimer’s disease, autoimmune encephalopathy, encephalitis and Parkinson’s disease. In the ongoing pilot investigation, ES-QuIC and EP-QuIC have been evaluated in parallel without an apparent reduction in specificity or sensitivity. We acknowledge the relatively limited sample size of the control population in our retrospective cohort. As parallel testing continues, larger prospective validation cohorts will be required to more precisely characterize specificity and the potential for false-positive results across the broader differential diagnosis of suspected CJD. Multicentre validation studies are warranted to determine whether this balance is reproducible across independent laboratories and more diverse patient populations.

Although there are currently no effective treatments available for CJD, the therapeutic landscape is evolving, with approaches aimed at reducing PrP expression or availability in the central nervous system, advancing into clinical trials (ION717; PRiSM) (Gentile et al., 2026; Raymond et al., 2019). A major challenge for these efforts is the lack of sensitive and reliable methods to detect and monitor disease over time, particularly in early-stage or genetically at-risk individuals. Clinical trials involving carriers of IPDs are well-positioned to monitor asymptomatic individuals throughout treatment regimens yet remain constrained by the limited sensitivity of existing assays, which do not consistently detect these cases. Given the growing interest in detecting clinically relevant prion pathogenesis in human-derived substrates, particularly following reports of positive signals in patient samples before clinical onset, ES-QuIC using the Dm PrP_23–230_ substrate represents a promising approach for IPD-sensitive prion detection, suitable for both longitudinal monitoring and trial readiness.

With declining autopsy rates and increasingly limited access to post-mortem confirmation, the importance of reliable ante-mortem diagnostic tools with high specificity rates will continue to grow. Earlier and more accurate diagnosis provides patients and their families with valuable time to process and prepare for disease progression, while also supporting clinicians in distinguishing prion diseases from other neurological conditions with overlapping features. Improved diagnostic clarity may also facilitate timely eligibility for emerging therapeutic trials. Continued innovation, rigorous validation, and harmonization of diagnostic practices will be essential to establishing a comprehensive and reliable framework for prion disease detection across its full clinical and molecular spectrum (Schwartz et al., 2019; Shimamura and Satoh, 2025).
